# Reviewed article: Batista JFC, Santos MAA, Lima SO. Trend of epidemiological indicators and factors associated with tuberculosis treatment dropout and death among street people in Brazil: an ecological and cross-sectional study, 2014-2022. Epidemiol Serv Saude. 2025:34;20240273

**DOI:** 10.1590/S2237-96222024v34e20240273.c

**Published:** 2025-04-14

**Authors:** Jesem Douglas Yamall Orellana

**Affiliations:** Leônidas e Maria Deane Institute Fiocruz Amazonas

**Table:** 

Rating	Excellent	Good	Average	Below average	Poor
Interest		✓			
Quality					✓
Originality			✓		
Overall				✓	

**Table:** 

Questionnaire	Yes	No	Not applicable
Does the manuscript contain new and significant information to justify publication?	✓		
Does the Abstract (Summary) clearly and accurately describe the content of the article?		✓	
Is the problem significant and concisely stated?	✓		
Are the methods described comprehensively?		✓	
Are the interpretations and conclusions justified by the results?	✓		
Is adequate reference made to other work in the field?	✓		

## Manuscript Structure:

Length of article is: Adequate

Number of tables is: Adequate

Number of figures is: Adequate

Please state any conflict(s) of interest that you have in relation to the review of this paper (state “none” if this is not applicable): None

